# Prevalence of Obstructive Sleep Apnea Syndrome and CPAP Adherence in the Elderly Chinese Population

**DOI:** 10.1371/journal.pone.0119829

**Published:** 2015-03-16

**Authors:** Susanna S. S. Ng, Tat-On Chan, Kin-Wang To, Ken K. P. Chan, Jenny Ngai, Alvin Tung, Fanny W. S. Ko, David S. C. Hui

**Affiliations:** Division of Respiratory Medicine, Department of Medicine and Therapeutics, The Chinese University of Hong Kong, Prince of Wales Hospital, Shatin, NT, Hong Kong; Charité - Universitätsmedizin Berlin, GERMANY

## Abstract

**Background:**

This study assessed the prevalence of obstructive sleep apnea syndrome (OSAS) and CPAP adherence in the elderly Chinese in Hong Kong.

**Methods:**

We conducted a sleep questionnaire survey among the elders aged ≥60 years in the community centres followed by level 3 home sleep study (Embletta). Subjects with an apnea hypopnea index (AHI) ≥ 15/hr alone and those with AHI ≥ 5/hr plus either cardiovascular risk factors or Epworth Sleepiness Score (ESS) ≥ 10 were offered CPAP treatment.

**Results:**

Altogether 819 subjects were interviewed with mean (SD) age of 73.9 (7.5) years, BMI 24.2 (3.6) kg/m^2^, neck circumference 34.9 (3.4) cm and ESS 6.6 (5.2). Daytime sleepiness was reported by 72.4%, snoring loudly 5.1% and witnessed apnea 4%. Among 234 subjects who underwent home sleep study, 156 (66.7%), 102 (43.6%), 70 (29.9%) and 45 (19.2%) had AHI ≥ 5, ≥ 10, ≥ 15 and ≥ 20/hr respectively, with the prevalence increasing with age and BMI. In the sample, 149 subjects (63.7%) were classified as having OSAS, as defined by an AHI ≥ 5/hr with associated symptoms, involving 81 men (74.3%) and 68 women (54.4%). Neck circumference and snoring frequency were the only positive independent factors associated with the AHI and the diagnosis of OSAS. Among 141 subjects who were offered CPAP treatment, 30 accepted CPAP prescription with improvement of ESS and cognitive function over 12 months with CPAP usage of 4.2 (2.2) h/night.

**Conclusion:**

This study showed a high prevalence of OSAS among the community elders in Hong Kong. Home CPAP acceptance was low but there was significant improvement of subjective sleepiness and cognitive function among those on CPAP treatment.

## Introduction

Sleep disturbances, such as daytime sleepiness and insomnia, are common among the elderly population[1]. Although sleep problems may be related to medical or psychiatric illness, sedating medications, primary sleep disorders such as sleep disordered breathing, periodic limb movement disorder and restless legs syndrome are also important factors[2].

Obstructive sleep apnea syndrome (OSAS) is a common disorder with prevalence rates of at least 4% among the middle-aged male Caucasians and Hong Kong Chinese populations[3–5]. Intermittent hypoxia and sympathetic surges, which characterize OSAS, can lead to oxidative stress, systemic inflammation, endothelial dysfunction[6–8], and result in sleep fragmentation, daytime sleepiness, impaired cognitive function and poor health status[9]. OSAS patients are at increased risk of cardiovascular morbidity and mortality including sudden death [10, 11], and are more prone to traffic accidents [12].

The prevalence of sleep disordered breathing (SDB) has been reported to be 20–81% in the elderly Caucasian populations[13]. In a French study, OSAS was diagnosed in 57% of the community-dwellers aged 68 years, 34% having a mild form with an apnea-hypopnea index (AHI) 15–30/hr whereas 23% had an AHI >30/hr[14]. Gender plays an important role in the pathogenesis of OSAS, which is more prevalent in men than in women [15]. The sex specific disparity of OSAS occurrence is likely attributable to a variety of factors, including hormonal influences and sex related differences in upper airway anatomy and body fat deposition. However, the prevalence of OSAS in the elderly community population in Asia including Hong Kong and its gender difference were unknown.

Nasal continuous positive airway pressure (CPAP) is a highly effective treatment for OSAS. A review reported positive effects of CPAP treatment in the elderly on sleep architecture, subjective daytime sleepiness, self-reported symptoms and nocturia [16]. However, the level of evidence for CPAP acceptance and compliance with treatment in elderly subjects is still insufficient based on previous publications with small clinical samples[17].

The primary objective of this study was to evaluate the prevalence of OSAS in the elderly subjects living in the community in Hong Kong. Our secondary objectives were to examine the factors predictive of the presence of OSAS in this population and assess CPAP acceptance and adherence, subjective sleepiness, cognitive function and health status at baseline and 12 months after treatment among the elderly with OSAS.

## Materials and Methods

The protocol for this trial and supporting TREND checklist are available as supporting information; see S1 TREND Checklist and S1 Protocol.

### Subjects

This prospective study recruited subjects from September 2007 to August 2010, from community centers in Hong Kong where senior citizens attended for social gatherings. Community centers were selected randomly for participation in the study and each center put up a poster to advertise the study during their group activities. Elderly subjects aged ≥ 60 years who were willing to join the study were requested to attend each center on designated days for interview. Informed written consent was obtained from each subject and the study was approved by the research ethics committee of the Chinese University of Hong Kong (Shatin, Hong Kong).

### Questionnaire

Apart from the usual demographic data, the sleep and health questionnaire (SHQ) and the Epworth sleepiness scale (ESS) were administered to the elderly subjects. The questionnaire was explained to the subjects and assistance was provided to read or further clarify the questions if necessary.

The SHQ is a modified version of the Specialised Centers of Research Sleep Questionnaire, and previously reported as a valid means of characterizing symptom distributing in population surveys of sleep apnea[18]. The questionnaire contained 16 questions, grouped into five factors (functional impact of sleepiness, self-reported breathing disturbances, roommate-observed breathing disturbances, driving impairment and insomnia) shown to be useful in predicting the occurrence of sleep apnea. Additional questions were added to document relevant medical history, current medications, the use of alcohol and the subjects’ sleeping habits, including the subjects’ usual sleep schedules.

The ESS questionnaire is specific to symptoms of daytime sleepiness and subjects are asked to score the likelihood of falling asleep in eight different situations with different levels of stimulation, adding up to a total score of 0–24[19]. The ESS was reported to be more discriminating than the Maintenance of Wakefulness Test and the Multiple Sleep Latency Tests as a test of daytime sleepiness[20]. Normal controls were found have scores of 5.9 ± 2.2 while subjects with OSAS had scores of 11.7 ± 4.6.

### Sleep monitoring

Subjects who had completed the questionnaires and consented for sleep study were invited to undergo a portable at-home sleep study. In the afternoon, subjects attended the pulmonary function laboratory to be fitted with the Embletta portable diagnostic system (PDS, Medcare, Iceland). Descriptions of the device and the validity of the methods used to collect and display data have been published previously among both local Chinese and other populations [21, 22]. It is a multi-channel screening tool that measures airflow through a nasal cannula connected to a pressure transducer, providing an AHI based on recording time. It also detects both respiratory and abdominal efforts through the effort sensor and can differentiate between obstructive and central events. The body position was detected by the built-in sensors. Respiratory events were scored when desaturations of at least 4% occurred in the absence of moving artifacts and irrespective of co-existing changes in snoring or heart rate. A hypopnea was defined as a decrease in airflow by 50% of baseline for at least 10 seconds. Data were included in the analysis if the total recorded evaluation time of 4 hrs or longer was obtained during the Embletta study.

According to the American Academy of Sleep Medicine (AASM) task force, diagnostic criteria for OSAS in adults include AHI ≥5/hr if patients have any of the following symptoms: loud snoring, breathing interruptions, waking with breath holding, gasping or choking, unintentional episodes during wakefulness, daytime sleepiness, unrefreshed sleep, fatigue or insomnia. Patients without the above symptoms but with AHI 15/hr or more are also diagnosed to have OSAS. [23] The result of home sleep study together with the symptoms derived from the questionnaires was used to calculate the disease prevalence.

As OSAS may increase the risk of cardiovascular mortality, all elderly subjects with AHI ≥ 15 or those with AHI ≥ 5 plus either cardiovascular risk factors or ESS score ≥ 10 received patient education program [24]. Subjects who agreed for CPAP therapy were offered basic CPAP education package consisting of a 10-min CPAP education programme by a respiratory nurse explaining the basic operation and care of the CPAP device and the mask, educational brochure on OSAS and CPAP treatment in Chinese, and careful mask fitting, and a short trial of CPAP therapy with the AutoSet CPAP device (Resmed, Sydney, Australia) for approximately 30 minutes for acclimatization in the afternoon[25, 26]. Attended CPAP titration was performed with the AutoSet auto-titrating device our hospital. Throughout the night and the next morning the nurses on duty would deal with any discomfort related to the CPAP treatment. The CPAP pressure for each patient was set at the minimum pressure needed to abolish snoring, obstructive respiratory events and airflow limitation for 95% of the night as determined by the overnight AutoSet CPAP titration study. The patients subsequently were followed up by physicians and nurses at the CPAP clinic in 1 month and 3 months later to deal with any problem with the CPAP device or mask fit [25, 26]. Elderly subjects who agreed for home CPAP treatment were prescribed nasal CPAP units with time clocks to assess objective compliance (run time). ESS, sleep apnea specific quality of life index (SAQLI)[27], and cognitive function tests were performed at baseline, 3 months, 6 months and 12 months after CPAP treatment. The follow up period finished in August 2011.

The SAQLI has 35 questions organized into four domains: daily functioning, social interactions, emotional functioning and symptoms with a fifth domain, treatment-related symptoms, to record the possible negative impacts of treatment. It contains items shown to be important to patients with OSAS and is designed as a measure of outcome in clinical trials in sleep apnea[27]. Cognitive function tests including trail-making, digit-symbol, digit-span and Stroop colour testing were performed to provide objective evidence for improvement in daytime function on CPAP treatment as in our previous study[26].

The trial was not registered before enrolment of subjects but performed later because the recommendation of CPAP therapy was standard treatment as suggested by AASM.[24] We confirmed that all ongoing and related trials for this intervention were registered.

There were three points of deviations from the original study protocol that was approved by the ethics committee. Firstly, the original protocol was designed to use a class IV portable sleep study device (ApneaLink sleep screener) for detection of subjects at risk of OSAS. We actually used a more superior, class III, home monitoring system, Embletta as it measures not only oxygen saturation, but also detects both respiratory and abdominal efforts through the effort sensor and can differentiate between obstructive and central events. Secondly, the original plan was to randomly select subjects to have home sleep study. However, most elderly subjects refused to participate in the sleep study as they were required to come to the pulmonary function laboratory for fitting the sleep study device due to long distance or walking disability. Therefore, we offered home sleep monitoring for those elderly subjects completed questionnaires and agreed to proceed. Lastly, we originally planned to offer CPAP treatment only for those with AHI ≥ 20/hr but we changed and offered for those with AHI ≥ 15/hr or those with AHI ≥ 5/hr plus either cardiovascular risk factors or ESS score ≥ 10 received patient education program due to the new published guidelines by AASM.[24]

### Statistical analysis

Data were given as means and standard deviations, unless otherwise stated. A *P*-value of <0.05 was considered significant. Stepwise multiple linear regression analysis was performed to examine the associations between AHI and demographics, neck circumference, smoking and alcoholic histories, CVS comorbidities, ESS and several questions asked in SHQ. Logarithmic transformation was conducted to AHI in order to tally with normality assumptions of the regression model. Logistic regression analysis was also performed to examine the associations between diagnosis of OSAS and several variables which were the same as that in stepwise multiple linear regression. The CPAP users and non-CPAP users were compared using an unpaired Student’s t-test and Mann-Witney *U*-test for variables measured on a continuous scale. Analysis of variance (ANOVA) with repeated measures was performed to assess changes in SAQLI and cognitive function tests after 3 months, 6 months and 12 months of nasal CPAP treatment among the CPAP users. A *P*-value of <0.05 was used to indicate differences between the groups that were statistically significant. All statistical analyses were conducted using the SPSS statistical software package (SPSS for Windows, version 12.0, SPSS, Chicago, IL).

## Results

**Fig 1:**
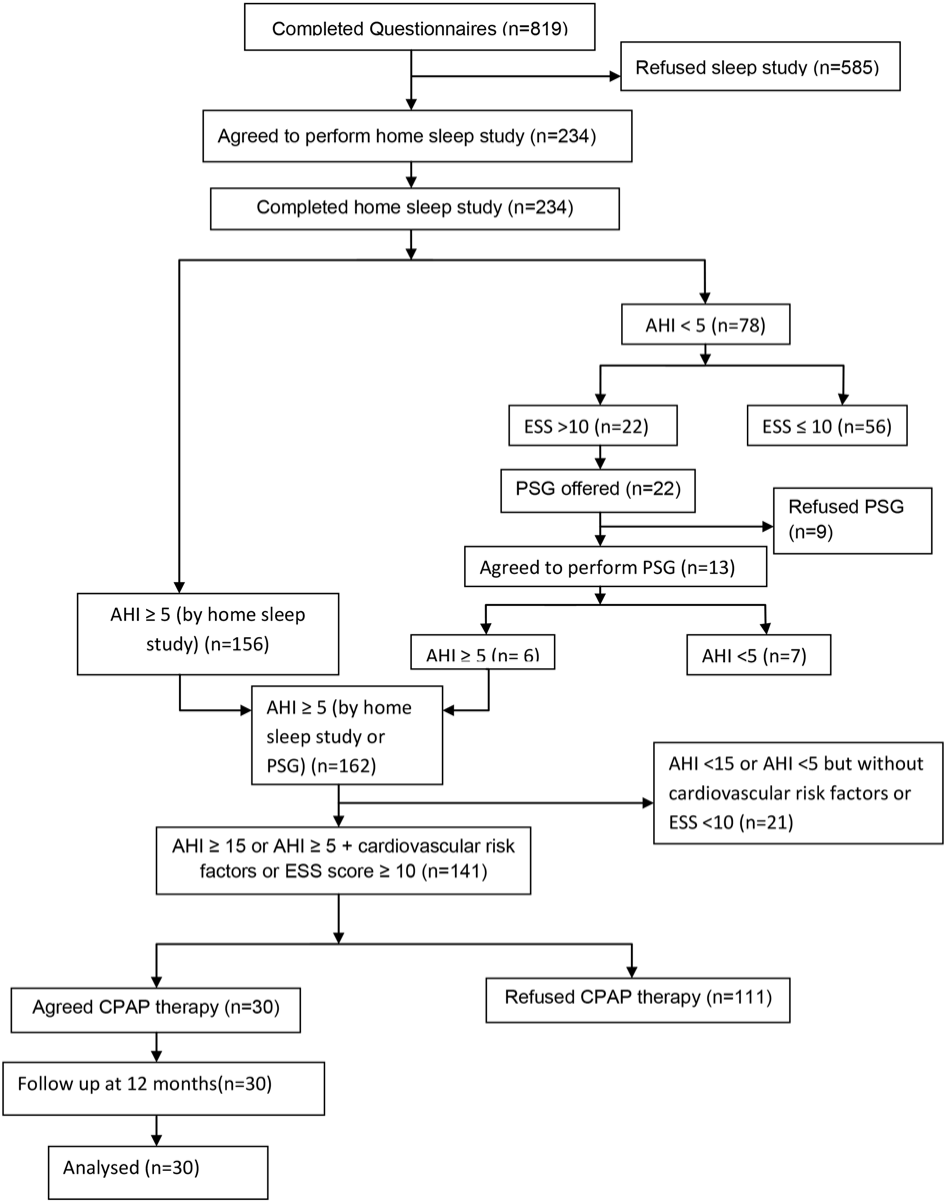
Study flow chart. AHI = apnea hypopnea index. CPAP = Continuous positive airway pressure. ESS = Epworth Sleepiness Score. PSG = polysomnography.

Altogether 819 subjects of Chinese ethnic background completed the SHQ (Table 1 and Fig. 1). Mean age was 73.9 ± 7.5 (SD) years, and the mean body mass index (BMI) was 24.2 ± 3.6 kg/m^2^. Compared to males, females had similar BMI but smaller neck circumference. More men were smokers and drinkers than women. The ESS was 6.6 ± 5.2 and there was no significant gender difference. Four hundred and fourteen subjects (50.5%) were hypertensive, 18.6% had diabetes mellitus, 7.5% had congestive heart failure, 6.4% had a history of cerebrovascular accident, and 4.7% had a history of ischemic heart disease.

**Table 1:** Clinical data of the studied population (N = 819) according to gender (Mean ± SD).

	Total	Men	Women
N (%)	819 (100%)	207 (25.3%)	612 (74.7%)
Age (years)	73.9 ± 7.5	73.4 ± 6.9	74.1 ± 7.6
BMI (kg/m^2^)	24.2 ± 3.6	24.3 ± 3.3	24.2 ± 3.7
BMI >23 (%)	498 (61.6%)	128 (62.7%)	370 (61.3%)
BMI >25 (%)	320 (39.8%)	84 (41.2%)	236 (39.3%)
NC (cm)	34.9 ± 3.4	37.7 ± 2.9	33.8 ± 2.9***
Non smoker (%)	696 (85.0%)	119 (57.5%)	577 (94.3%)***
Non drinker (%)	630 (76.9%)	121 (58.5%)	509 (83.2%)***
ESS	6.6 ± 5.2	7.0 ± 5.2	6.5 ± 5.2

Comparison between women and men based on independent samples *t*-test or chi-square test, as appropriate.

* *p*≤0.05;

** *p*≤0.01;

*** *p*≤0.001.

NC = Neck circumference; BMI = Body mass index; ESS = Epworth Sleepiness Score.

Results from selected SHQ and more specific symptoms predicting OSAS are listed in Table 2. Because the majority of our subjects (89%) did not drive vehicle, questions related to driving impairment from the SHQ were not included in the data analysis. Among 592 subjects (72.4%) who complained of daytime sleepiness, 185 subjects (31.5%), 144 females (33.3%) and 41 males (26.5%), always experienced the symptom. Of the 333 subjects (40.7%) who reported a history of snoring, 17 subjects (5.1%), 8 females (3.7%) and 9 males (7.7%), reported extremely loud snoring. Nineteen subjects (2.3%) and 33 (4%) had a history of witnessed apnea and observed choking, respectively, whereas 16 (2%) reported a combination of snoring plus witnessed apnea.

**Table 2:** Responses to selected questions from the Sleep & Health Questionnaire. ^[^ ^18^ ^]^

n = 819 (Male = 207)	Mild	Moderate	Severe
Impaired performance ability	41.5%	8.5%	8.7%
Sleepiness interfered with daily tasks	10.2%	3.4%	3.8%
Impaired energy level	39.2%	8.0%	9.4%
Daytime sleepiness	41.1%	8.5%	22.8%
Snoring intensity (past month)	33.0%	8.2%	5.1%
Snoring frequency (past month)	24.6%	6.7%	19.3%
Frequent awakenings	38.3%	8.6%	20.7%
Difficulty falling asleep	48.4%	7.8%	9.9%
Observed awakenings	6.6%	0.7%	0.8%
Observed choking	2.8%	1.1%	0.7%
Observed apnea	1.7%	0.4%	0.6%

The responses to the questionnaire utilized either a 5-point frequency scale ("never", "rarely", "sometimes", "frequently" and "almost always"); or by the use of a 6—point Likert scale which graded the severity of the symptoms. The responses were categorized into mild, moderate and severe as follows for the purpose of data analysis^18,29,30^:

*6-point Likert scale*: 1–2 points (not affected); 3–4 points (mild); 5 points (moderate); 6 points (severe)

*5-point frequency scale*: never (not affected); rarely or < once/week(mild); sometimes or 1–2/week (mild); frequently or 3–4/week(moderate); almost always or 5–7/week (severe)

*Responses to snoring intensity*:

0 (not affected); 1–2 points (mild); 3 points (moderate); 4 points (severe)

### Home sleep studies

Following the questionnaires, 234 elderly subjects consented to have overnight home sleep monitoring. The baseline characteristics were similar to the original sample population, except that they were younger and had higher ESS score (Table 3). There was measurement failure involving 18 of the 246 home sleep studies; but, subsequently, the studies were repeated, producing interpretable recordings. The mean AHI for those with home sleep studies was 12.7±13.1/hour, while mean AHI for female subjects was 9.15±9.5/hour and male subjects 16.8±15.3/hour. The mean duration of objective snoring was 12.5±12% of the night whereas 73 (31.2%) snored for ≥10% of the night.

**Table 3:** Baseline characteristics of subjects with and without Embletta home sleep study (mean ± SD).

	With Embletta (*n* = 234)	Without Embletta (*n* = 585)
Age (year)	70.6 ± 7.1	75.3 ± 7.2
BMI (kg/m^2^)	24.5 ± 3.3	24.1 ± 3.7
Neck circumference (cm)	35.1 ± 3.2	34.8 ± 3.5
ESS	7.5 ± 5.5	6.3 ± 5.1
Self-reported sleep time (h)	7.3 ± 4.1	7.3 ± 1.6

BMI = Body mass index; ESS = Epworth Sleepiness Scale.

Based on the sleep studies, 156 (66.7%), 102 (43.6%), 70 (29.9%) and 45 (19.2%) subjects had AHI ≥ 5, ≥ 10, ≥ 15 and ≥ 20/hour of sleep respectively. The 95% Confidence Intervals (CI) for the frequency of elderly in the four AHI categories (≥5, ≥10, ≥15 and ≥20/hour) were 61–73%, 37–50%, 24–36% and 14–24% respectively. In the sample, 149 subjects (63.7%) subjects were classified as having OSAS, (CI 45.2–72.3%) as defined by an AHI of ≥15/hr, or AHI ≥5/hr with associated symptoms, involving 81 men (74.3%) and 68 women (54.4%). Their mean ESS was 8.1 ± 5.4.

**Fig 2:**
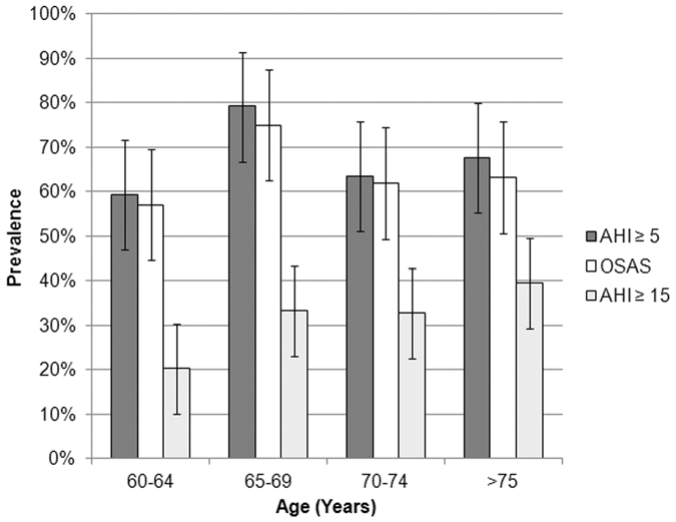
OSAS prevalence estimates with 95% confidence intervals are illustrated based upon AHI cutoffs of 5 and 15, and diagnosis of OSAS according to age quartile.

With this definition, OSAS prevalence is more prevalent at the age of 65–69 yrs with 75% of subjects fitting into the diagnosis (CI 62.3–87.7%). After that, the prevalence is slightly reduced but still 63.2% of subjects were found to be affected (CI 57.5–69.9%) (Fig. 2).

**Fig 3:**
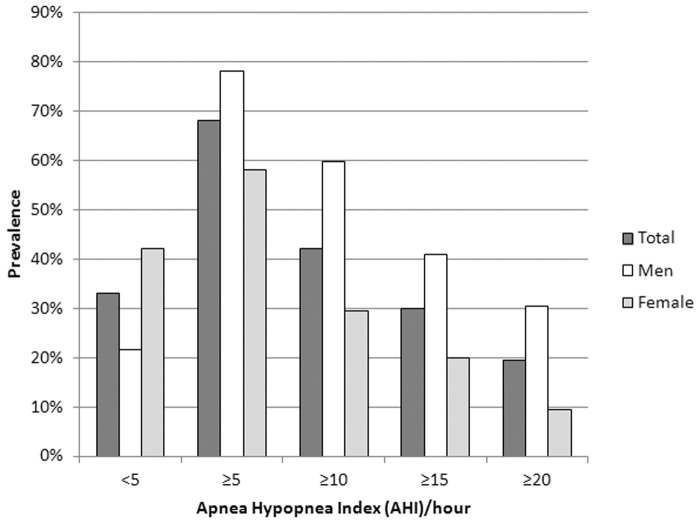
OSAS prevalence illustrated upon gender according to apnea hypopnea index (AHI) quartile.

Table 4 compares subjects with AHI <15 or ≥ 15/hr. Subjects with higher AHI had greater neck circumference and that were more than those in female subjects who had high AHI. Moreover, male with higher AHI had higher BMI than those male subjects with low AHI, although no significant difference among the female subjects was observed. Within the high AHI group, hypertension was more frequent in women than in men. Together with Fig. 3, the result shows the prevalence of sleep disordered breathing in various AHI cutoff values, females had less severe AHI. Only 20% of women had AHI ≥ 15/hour. No differences in subjective total sleep time, ESS, minimal and mean oxygen saturation were found between men and women with high AHI.

**Table 4:** Clinical data for the samples (n = 234) with AHI less or equal to versus those with AHI greater than 15 events per hour according to gender (mean ± SD).

	AHI <15/hr			AHI >15/hr		
	Total (n = 164)	Men (n = 64)	Women (n = 100)	Total (n = 70)	Men (n = 45)	Women (n = 25)
Age (years)	70.3 ± 7.2	73.1 ± 6.8	68.4 ± 6.9	71.3 ± 6.8	72.1 ± 6.9	70.0 ± 6.5
BMI (kg/m2)	24.3 ± 3.4	23.6 ± 3.0	24.7 ± 3.6	25.1 ± 3.2	24.9 ± 3.1 ^+^	25.4 ± 3.4
BMI >23 (%)	109 (66.5%)	36 (56.3%)	73 (73.0%)	51 (72.9%)	32 (71.1%)	19 (76.0%)
BMI > 25 (%)	70 (43.2%)	22 (34.4%)	48 (49.0%)	40 (57.1%)	23 (51.1%)	17 (68.0%)
Neck circumference (cm)	34.6 ± 3.1	36.7 ± 2.8	33.2 ± 2.5	36.2 ± 3.0 ***	37.6 ± 2.2	33.5 ± 2.3^§§§^
Congestive heart failure (%)	6 (3.7%)	3 (4.7%)	3 (3.0%)	5 (7.1%)	3 (6.7%)	2 (8.0%)
Diabetes (%)	32 (19.5%)	14 (21.9%)	18 (18.0%)	10 (14.3%)	5 (11.1%)	5 (20.0%)
Hypertension (%)	78 (47.6%)	36 (56.3%)	42 (42.0%)	33 (47.1%)	17 (37.8%)	16 (64.0%) ^§^
Cerebrovascular accident (%)	7 (4.3%)	3 (4.7%)	4 (4.0%)	6 (8.6%)	5 (11.1%)	1 (4.0%)
Ischemic heart disease (%)	8 (4.9%)	6 (9.4%)	2 (2.0%)	4 (5.7%)	3 (6.7%)	1 (4.0%)
Subjective total sleep time (min)	423.2 ± 61.2	422.1 ± 63.6	423.9 ± 60.0	426.1 ± 70.3	419.7 ± 72.1	437.7 ± 66.8
ESS	7.4 ± 5.5	6.5 ± 4.5	8.0 ± 6.1	7.6 ± 5.3	7.4 ± 5.3	8.0 ± 5.4
AHI (events/hour)	5.8 ± 4.1	6.9 ± 4.4	5.3 ± 3.8	28.8 ± 12.7 ***	31.1 ± 13.7 ^+++^	24.7 ± 9.8 ^###^ ^,^ ^§^
Mean SaO2 (%)	95.3 ± 1.6	95.4 ± 1.5	95.3 ± 1.6	94.4 ± 2.0 ***	94.4 ± 2.1 ^++^	94.3 ± 1.6 ^##^
Minimal SaO2 (%)	84.2 ± 6.0	83.3 ± 7.4	84.8 ± 4.9	77.2 ± 8.8 ***	77.9 ± 8.6 ^+++^	76.1 ± 9.0 ^##^

Comparison between total subjects with AHI>15 and AHI <15:

*p≤0.05;

**p≤0.01;

***p≤0.001.

Comparison between men with AHI>15 and AHI <15:

^+^ *p*≤0.05;

^++^ *p*≤0.01;

^+++^ *p*≤0.001.

Comparison between women with AHI >15 and AHI <15:

^#^ *p*≤0.05;

^##^ *p*≤0.01;

^###^ *p*≤0.001.

Comparison between women and men with AHI >15:

^§^ *p*≤0.05;

^§§^ *p*≤0.01;

^§§§^ *p*≤0.001.

BMI = Body Mass Index; NC = neck circumference; ESS = Epworth Sleepiness Score; AHI = Apnea-Hypopnea Index.

### Factors correlating with AHI and OSAS

Neck circumference and snoring frequency were found to be the two positive and independent factors associated with both the severity of AHI (*P* <0.001 and *p* = 0.003 respectively), and the diagnosis of OSAS (*p* = 0.006 and *p* = 0.001). (Tables 5 & 6)

Neck circumference was positively correlated with AHI (odds ratio = 1.26) with one centimeter of increase in neck circumference associating with 0.113 increase in AHI (CI 0.06–0.165). Similar positive correlation was identified with the snoring frequency (odds ratio 1.59) with one point increment in the frequency scale of snoring associated with 0.15 increases in AHI.

**Table 5:** Stepwise multiple linear regression results on the association with AHI.

	Coefficients		95% CI for beta		
	beta	SE	Lower Bound	Upper Bound	*P* value
Neck circumference	0.113	0.027	0.060	0.165	<0.001*
Snoring frequency^§^	0.152	0.050	0.052	0.251	0.003*

Number of sleep study tested subjects = 234.

^§^5-point frequency scale.

* AHI was logarithmic transformed.

**Table 6:** Logistic regression analysis on the association between patients suffering from OSAS and several independent variables.

	odds ratio (OR)	95% CI for odds ratio (OR)		
		Lower Bound	Upper Bound	*P* value
Subject's age	1.012	0.952	1.076	0.694
BMI	0.907	0.803	1.024	0.116
Neck circumference	1.256	1.066	1.479	0.006*
ESS	0.974	0.887	1.069	0.578
Performance ability	1.363	0.992	1.873	0.056
Interference with daily tasks	1.014	0.705	1.460	0.939
Daytime sleepiness^§^	1.048	0.762	1.441	0.772
Snoring frequency^§^	1.589	1.195	2.113	0.001*
Frequent awakenings^§^	1.027	0.767	1.374	0.860
Difficulty falling asleep^§^	0.832	0.590	1.173	0.293
Observed awakenings^§^	0.849	0.362	1.989	0.706
Observed choking^§^	1.129	0.429	2.965	0.806

Number of sleep study tested subjects = 234.

^§^5-point frequency scale.

BMI = Body mass index; ESS = Epworth Sleepiness Scale; AHI = Apnea Hypopnea Index; CVS = cardiovascular disease.

### CPAP treatment outcome

Among 78 subjects with AHI <5/hr in home sleep studies, 22 sleepy subjects (i.e. ESS >10) were invited to undergo full PSG in our hospital for confirmation of OSAS. Only 13 (59%) subjects agreed to undergo PSG. Six subjects were confirmed to have OSAS in subsequent full PSG with AHI ≥ 5/hr. Together with those diagnosed to have OSAS by home sleep studies, 141 subjects who had AHI ≥ 15/hr or AHI ≥ 5/hr plus cardiovascular risk factors or AHI ≥ 5/hr plus ESS score ≥ 10 were offered CPAP titration with the AutoSet device after receiving a basic CPAP education package[25, 26]. The mean (SD) of CPAP pressure titrated was 12.0 (1.4) cmH2O. Only 30 elderly subjects accepted home CPAP treatment whereas 59% of them adhered to treatment at 4.6 (2.1) hrs/night at 3 months, 56% at 4.6 (2.2) hrs/night at 6 months, and 44% at 4.2 (2.2) hrs/night at 12months. ESS decreased from 9.2 (5.5) to 7.1 (5.6) at 12 months, *p* = 0.016.

Comparisons between those who accepted home CPAP (n = 30) and those who refused (n = 111) are shown in Table 7. The CPAP users had significantly higher BMI, more severe SDB (as reflected by a higher AHI, lower minimum SaO2 and mean SaO2), but there was no significant difference in baseline ESS. There was significant improvement of digit symbol, trail A, trail B, stroop colour and ESS among the CPAP users but no significant change in SAQLI and digit span at 12 months (Table 8).

**Table 7:** Comparisons between subjects who accepted and those who refused home CPAP treatment with variables expressed as median (IQR).

	CPAP users (n = 30)	Refused CPAP (n = 111)	P value
Age	67 (9)	68 (11)	0.16
BMI	26.8 (3.7)	25.6 (3.0)	<0.001
Neck circumference	36 (3.5)	36 (5.5)	0.04
AHI (Home Embletta / PSG)	16.8 (14.2)	14.6 (19.1)	0.02
MinSaO2	79 (14)	79 (13)	0.04
Mean SaO2	93.6 (2.4)	95 (2)	<0.001
CPAP pressure titrated	12 (1.8)	12 (1)	0.34
ESS	9 (8)	10 (6)	0.77

ESS, Epworth sleepiness scale; IQR, interquartile range.

**Table 8:** Changes in sleep apnea-specific quality of life index (SAQLI), cognitive function tests and ESS among CPAP users (n = 30).

	Baseline	3 months	6 months	12months	*P* values (repeated measures)
Total SAQLI score	5.0 (0.9)	5.2 (1.0)	5.3 (0.9)	5.4 (0.9)	0.106
Digit span	14.8 (3.8)	15 (3.3)	15.7 (3.9)	15.4 (4.2)	0.285
Digit Symbol	30.4 (12.2)	34.7 (14.3)	35.9 (14.1)	35.7 (15.1)	<0.001
Trail A	64.7 (23.7)	54.4 (23.2)	54.8 (25.5)	53 (23.9)	<0.001
Trail B	101.9 (53)	89.9 (57.7)	89.1 (51.6)	85.8 (41.3)	0.004
Stroop colour	60.2 (17.7)	64.2 (17.9)	66.4 (15.8)	65 (17.6)	0.001
ESS	9.2 (5.5)	7.0 (4.9)	7.2 (5.6)	7.1 (5.6)	0.003

The trail-making test estimated the minimum time required to connect a structured number sequence and the lower the score, the better the performance. The digit symbol and span tests involved the immediate memory and recall of number sequences while the stroop colour test evaluated the correct matching of colour and their corresponding characters. For the stroop colour, digit symbol and span tests, a higher score indicated superior performance.

ESS, Epworth sleepiness score.

Among 111 subjects who refused CPAP therapy, 38% had poor insight about the significance of untreated OSAS, 32.4% found it inconvenient to use, 26.8% could not sleep well overnight with CPAP, 21.1% complained of mask discomfort, 2.8% had exacerbated symptoms of allergic rhinitis, 1.4% had no room to place CPAP and 1.4% could not afford to purchase the CPAP machine.

## Discussion

To the best of our knowledge, this has been the first prospective epidemiological study examining a large cohort of community-based elderly subjects in Asia. This study has shown a high prevalence of sleep symptoms, such as daytime sleepiness (72.4%), frequent awakenings (67.6%), sleep-onset insomnia (66.1%) among our elderly subjects. Daytime sleepiness was the most common sleep problem among these subjects and the cause might be multifactorial. The group ESS, regardless of the AHI, was similar to the ESS in the normal population[28]. Sforza *et al* reported similar findings in their community elderly study and showed that those with AHI ≥ 15/hr were not sleepy by the ESS score[14].

This study has shown a high prevalence of significant OSAS (63.7%) among the sample tested in a group of community-based elderly subjects in Hong Kong. It is much higher than those reported among the middle-aged male Chinese populations in Hong Kong despite similar BMI [5, 29, 30]. Although the prevalence of the disorder was found to be lower after the age of 70 yrs, 63.2% of the elderly population over 75 years suffered from the disease.

Young *et al* [31] reported an increase in SDB prevalence with increasing 10-year age groups, with the association with an increase in the odds of having an AHI of 15 or greater by 24%. In our study, neck circumference and snoring frequency had a positive and independent association with AHI, whereas traditional risk factors, such as BMI, snoring intensity and ESS, were not after multiple regression analysis. The finding was consistent with previous studies.[31, 32]

The ratio of men to women with OSAS in this study appears to be considerably higher than other community studies.[3, 29, 33] The reason of this discrepancy is not clear. Men with OSAS have a higher neck circumference than female patients although there was no significant difference when compared with non-OSAS subjects in each individual sex in our study. Moreover, female OSAS patients tend to suffer from hypertension more than the male group. Sforza *et al* also compared women with and without OSAS and showed no significant differences in clinical or anthropometric measures[14]. The presence of hypertension was significantly associated with OSAS risk in women with the odds ratio of 1.52. All these data suggest that the occurrence of OSAS is associated with hypertension in the elderly women.

Recently, a study of CPAP use in elderly OSAS patients in Spain has shown that untreated severe OSAS is associated with cardiovascular death in the elderly, and adequate CPAP treatment may reduce this risk [34]. Nevertheless, 78.7% of our subjects with OSAS refused CPAP treatment while many of them did not appear to have a clear understanding of the health consequences of OSAS. Of the 30 subjects who accepted CPAP therapy, only 44% of them adhered to the treatment with the nightly use of 4.2 (2.2) hours. There was significant improvement of ESS, digit symbol, trail A and B, and stroop colour after 12 months of CPAP treatment. These CPAP users clearly had higher BMI and neck circumference, and more severe SDB, as reflected by a higher AHI than those who refused CPAP treatment. A limited number of studies have examined interventions to improve CPAP use in older adults, including cognitive behavior therapy intervention, intensive support intervention and monthly home visits [35, 36].

There are several limitations in this study. First, the elderly subjects were chosen from the community centers and it is possible that those with more symptoms would be more eager to join the study. There were more females than males, although this is the characteristic in the elderly centers nowadays. In addition, despite our attempt to minimize selection bias (by distributing two consent forms for participation in the sleep questionnaire and the portable home sleep study explaining the purpose of the project without details of the questionnaire), those who participated in the home sleep study were younger, with slightly higher ESS. Moreover, patients who had chosen to use CPAP suffered from more severe OSAS and lower mean SaO2 as shown in Table 6, although there was no difference in the severity of sleepiness between the two groups.

In summary, this study has shown a high prevalence of significant sleep disordered breathing and OSAS among subjects tested from a large community cohort of elderly Chinese in Hong Kong. The prevalence of OSAS based on home sleep study is up to 63.7% among those subjects tested and is higher than that in the middle aged population. Neck circumference was the positive independent predictor of AHI whereas ESS, BMI and snoring intensity could not identify subjects with SDB. Home CPAP acceptance was low but there was significant improvement of subjective sleepiness and cognitive function among those adhered to CPAP treatment. Further studies in determining the factors associated with CPAP compliance in the elderly and the interventions to promote their compliance are needed.

## Supporting Information

S1 TREND Checklist (PDF)

S1 Protocol Original protocol of the study. (DOC)
