# Supplementary material for: Prevalence of Obstructive Sleep Apnea Syndrome and CPAP Adherence in the Elderly Chinese Population
Source: PLoS One. 2015 Mar 16;10(3):e0119829. doi: 10.1371/journal.pone.0119829 (PMC4361659; doi:10.1371/journal.pone.0119829)
Supplement: S1 Protocol — (DOC) [file pone.0119829.s002.doc]

Principal investigator: David SC Hui

Co-investigators: Susanna SS Ng, Fanny WS Ko, Kin-Wang To

**Project Title**

Sleep-disordered breathing and continuous positive airway pressure compliance in the elderly Chinese living in Hong Kong.

**Introduction and background**

Obstructive sleep apnea syndrome (OSAS) is a common form of sleep-disordered breathing (SDB) characterized by repetitive episodes of cessation of breathing during sleep due to upper airway collapse. It causes sleep fragmentation, disabling daytime sleepiness, impaired cognitive function and poor quality of life.^1^ In addition, OSAS is associated with non-fatal and fatal cardiovascular consequences including sudden death,^2,3^ in addition to an increased risk of road traffic accidents.^4^

OSAS is equally common among the middle-aged male Caucasian and Hong Kong (HK) Chinese populations with a prevalence of at least 4%.^5,6,7^ The prevalence and severity of OSAS tend to increase through adult life, peaking in the late fifties to mid sixties, after which it fails to increase or increase.^8,9^ Ancoli-Isreal et al.^10^ reported prevalence rates of SDB in a group aged 65-95 years to be 70% for men and 56% for women, at least double those reported for middle-aged cohorts.^11^ However, the prevalence of OSAS in the elderly in Southeast Asia is unknown.

Sleep disturbances, such as daytime sleepiness, SDB and non-respiratory causes including insomnia, restless leg syndrome are common in the elderly.^12^ The deterioration in nocturnal sleep quality with increasing age could be related to the quantitative decline in the amount of deep or slow wave sleep (stages 3 and 4) and an increase in the proportion of light sleep (stages 1 and 2). There are also more frequent and sometimes prolonged nocturnal awakenings, leading to a decline in sleep efficiency (total time asleep as percentage of time in bed).^13^ However, the disruption of sleep architecture can be exaggerated by the presence of OSAS. The associated hypersomnolence can exacerbate the normal tendency in older people to sleep during the day, affecting their functional independence.

The specific impact of OSAS on mortality in the elderly population has been examined. A previous study has found a significant association between OSAS and mortality in women in a study of SDB in nursing home residents.^14^ The frequency of respiratory disturbances during sleep positively correlated with mortality, and women with OSAS had a greater risk (66 versus 41%) of dying in their sleep. Subsequent study about mortality in the elderly people with SDB established a significantly reduced survival in those with a respiratory disturbance index (RDI) of at least 30 per hour, but RDI was not an independent predictor of death.^15^

Worldwide the population is aging due to both lower birth rates and improved health care particularly in the developed countries. Life expectancy has increased greatly in many parts of the world along with improvement in nutrition, sanitation and economic factors.^16^ The greater longevity has resulted in more senior citizens in the population. While OSAS produces large negative impacts on health and quality of life, the recognition is not always straightforward because of the presence of co-morbidities and normal age-related changes in sleep quality and behavior. Once OSAS is suspected, it is not only relatively easy to diagnose, but usually responds well to treatment with CPAP. Therefore, a study of the prevalence and severity of OSAS in this age group in Hong Kong, and its treatment acceptance and compliance will surely provide important data for our health care planning.

**Aims of study**

1. We aim to evaluate the prevalence rates of SDB, OSAS and other sleep disturbances, such as restless leg syndrome (RLS), in the elderly subjects living in the community in HK.
2. In addition, we will examine the factors which are predictive of the presence of SDB in this population, and
3. Assess the CPAP acceptance, compliance, and treatment outcome of those with significant OSAS (AHI>=20/hr + ESS >10).^10^

**Dates for commencement and completion of the project**

Date of commencement: January 1, 2007

Date of completion of recruitment and polysomnography:

Date of completion of data analysis and reporting

**Study design**

A prospective epidemiological study.

**Research or study plan**

At least 1000 subjects will be recruited from the community elderly centers and hostels in Hong Kong. There are totally 114 neighbourhood elderly centers, 60 social centres for elderly, 40 district elderly community centers and 113 hostels for elderly in Hong Kong. All these centers are run by the government or charity organizations. The neigbourhood elderly centers for elderly, and district elderly centers are places that older people of the community can gather for various social activities. For hostels for the elderly, they are for older people who can take care (eg cooking and bathing independently) of themselves to liver together. Each of the neighbourhood elderly centers, social centre for elderly, district elderly community centers has roughly 200 to 600 elderly aged 60 years or above registered with the center. These centers and hostels for elderly will be selected randomly for this study with equal distribution in Hong Kong, Kowloon and the New Territories region.

Invitation letters will be sent to the centers for recruiting participants to join the study. Our research assistant and medical officers will visit the elderly center for the study. Subjects aged over 60 years old will be recruited. A questionnaire will be administered and information regarding their demographic data, the Sleep and Health Questionnaire (SHQ), the Epworth sleepiness scale (ESS) and restless leg symptoms will be obtained. The height, weight, baseline systolic and diastolic blood pressure will be measured.

The SHQ is a modified version of the Specialised Centres of Research Sleep Questionnaire, reported to be a valid means of characterizing symptom distribution in population surveys of sleep apnea.^17^ The SHQ contains 16 questions grouped into five factors (functional impact of sleepiness, self-reported breathing disturbances, roommates-observed breathing disturbances, driving impairment and insomnia) and is useful in predicting the occurrence of sleep apnea. Most responses to the questionnaire used either a five-point frequency scale (never, rarely, sometimes, frequently, and always) or a six-point Likert scale that graded symptom severity. These questions were categorized into mild, moderate, and severe for the purpose of data analysis. On the six-point Likert scale, one to two points indicated not affected; three to four points, mild; five points, moderate; and six points, severe. On the five point frequency scale, never indicated not affected; rarely or sometimes, mild; frequently, moderate; and always, severe. Responses to snoring intensity of zero points indicated not affected; one to two points, mild; three points, moderate; and four points, severe.

The ESS is a questionnaire specific to symptoms of daytime sleepiness and the subjects are asked to score the likelihood of falling asleep in eight different situations with different levels of stimulation, adding up to a total score of 0-24.^18^

*Syndrome Definitions for the Purpose of This Study*

RLS is a disorder characterized by disagreeable leg sensations that usually occur before sleep onset, causing an almost irresistible urge to move the legs. As minimal criteria for diagnosis^19^ the following four features are required: (1) desire to move the extremities, often associated with paresthesias and/or dysesthesias; (2) motor restlessness; (3) worsening of symptoms at rest, with at least temporary relief by activity; and (4) worsening of symptoms in the evening or at night.

OSAS is estimated by any of the following symptoms: snoring intensity (extremely loud indicates severe)^17^, observed choking^17^, witnessed apnea,^17,20^ snoring and witnessed apnea,^21^ disruptive snoring,^17,22^ and disruptive snoring and witnessed apnea.^22^

We aim to recruit a total of 1000 subjects and it is estimated we can recruit 50 subjects from each center and we thus need a total of 20 centers to participate in our study.

A portable sleep study device (ApneaLink sleep screener) for detection of patients at risk of OSAS will be validated. The ApneaLink is a double-channel, portable device using a nasal pressure transducer to measure apnea hypopnea index, flow limitation, and snoring, together with oximetry to monitor oxygen saturation during sleep. ApneaLink recordings will be scored with the computer-based automatic scoring system provided with the equipment. Nocturnal polysomnography (PSG) and ApneaLink recordings will be conducted simultaneously on 20 subjects presenting with symptoms of OSAS. Subjects will be assigned to hospital administration and are monitored consecutively. Records of PSG and ApneaLink will be analyzed in double-blind fashion. PSG records will be scored by 30-s epochs following the American Sleep Disorders Association standards for sleep states and stages and for sleep-related events, including sleep apneas, hypopneas, and periodic leg movements. Following independent scoring, the Apnea-hypopnea index based on each PSG and each ApneaLink analysis will be compared to determine their correlation by Pearson analysis. With the PSG used as the gold standard diagnostic method, the degree of error for each variable with the ApneaLink will be calculated. The results of this validation study will show whether ApneaLink could be helpful to general practitioners, clinicians, and epidemiologists as a low-cost screening device for subjects with OSAS and habitual snoring.

Three hundred elderly in the community who have completed the questionnaire survey and consented for sleep study will be randomly selected to have home sleep study. Every subject will be assigned a specific number on the consent form and randomization for sleep study will be performed by computer software using the number assigned. In the afternoon, subjects will attend the pulmonary function laboratory for the fitting of the ApeaLink device.. Subjects will sleep at home with the device attached and recorded the time they retire to bed and upon awakening. In the morning the device will be removed and the sleep data will be downloaded to a computer.

Data will be printed using the ApneaLink software program, and analysed by a single-blinded experienced scorer. Sleep time will be taken as the subjects’ report of time between bedtime and waking time.. The respiratory disturbance index (RDI), the number of respiratory disturbances per hour of estimated sleep, will be thus calculated for each subject. Significant SDB in elderly will be defined as RDI >/ 20/h of sleep plus self-reported daytime sleepiness.^10^

As SDB may increase the cardiovascular risk, all subjects with RDI >/20/h on ApneaLink study will be invited to undergo hospital-based PSG (Healthdyne Alice 4, Atlanta, GA, USA) with airflow measured by a nasal pressure transducer (PTAF 2, Pro-Tech, Woodinville, WA, USA) for confirmation of their sleep apnea status. Those with AHI >/ 20/hr on PSG will be offered a basic CPAP education package as described in our previous studies.^23^ Basically, the package consists of the following components: a 10-min CPAP education programme by a respiratory nurse explaining the basic operation and care of the CPAP device and the mask, educational brochure on OSA and CPAP treatment in Chinese, and careful mask fitting from a wide range of selection, and a short trial of CPAP therapy with the AutoSet CPAP device (Resmed, Sydney, Australia) for approximately 30min for acclimatization in the afternoon. Attended CPAP titration will be performed with the AutoSet auto-titrating device in our hospital. Throughout the night and the next morning the nurses on duty would deal with any discomfort related to the CPAP treatment. The CPAP pressure for each patient will be set at the minimal pressure needed to abolish snoring, obstructive respiratory events and airflow limitation for 95% of the night as determined by the overnight AutoSet CPAP titration study. The patients subsequently will be followed up by physicians and nurses at the CPAP clinic in 1 month and 3 months later to deal with any problem with the CPAP device or mask fit. Subjects who agreed for home CPAP treatment will be prescribed nasal CPAP units with time clocks to assess objective compliance (run time). ESS, sleep apnea-specific quality of life index (SAQLI),^24^ and cognitive function tests will be performed at baseline and at 3 months after CPAP treatment.

*Sleep apnea-specific quality of life index and cognitive function tests*

The SAQLI has 35 questions organized into four domains: daily functioning, social interactions, emotional functioning and symptoms with a fifth domain, treatment-related symptoms, to record the possible negative impacts of treatment. It contains items shown to be important to patients with sleep apnoea and is designed as a measure of outcome in clinical trials in sleep apnoea.^24^

Cognitive function tests including trail-making, digit-symbol, digit-span and Stroop colour testing will be performed to provide objective evidence for improvement in daytime function on CPAP treatment as in our previous study. ^23^ The trail-making test estimates the minimum time required to connect a structured number sequence and the lower the score, the better the performance. The digit symbol and span tests involve the immediate memory and recall of number sequences while the stroop colour test evaluates the correct matching of colour and their corresponding characters. For the stroop colour, digit symbol and span tests, a higher score indicate superior

performance.^23^

**Data analysis**

Data will be given as mean and standard deviation. Statistical significance will be assessed by T-test, ANOVA or chi-square test as appropriate. The association of several variables (such as age, BMI, neck circumference, ESS and SHQ

responses) versus RDI and versus objective snoring % will be evaluated using Pearson correlation analysis. Multiple linear regression analysis will be performed to

look for independent factors associated with either RDI or objective snoring %. Wilcoxon signedranks tests will be performed to assess changes in SAQLI and cognitive function tests after 3 months of nasal CPAP treatment among the CPAP users. A *p*value of <0.05 was used to indicate differences between the groups that were statistically significant. Data analysis will be performed with a commercially

available statistical analysis software package (SPSS 11.5 for Windows, SPSS Inc., Chicago, IL, USA).

Ethics committee approval will be obtained from the Chinese University of Hong Kong before commencement of the study and each subject will be required to give informed written consent.

Reference:

Engleman HM, Douglas NJ. Sleepiness, cognitive function, and quality of life in obstructive sleep apnoea/hypopnoea syndrome. *Thorax* 2004; **59**: 618-22.

[Marin JM](http://www.ncbi.nlm.nih.gov/entrez/query.fcgi?db=pubmed&cmd=Search&term="Marin+JM"%5BAuthor%5D), [Carrizo SJ](http://www.ncbi.nlm.nih.gov/entrez/query.fcgi?db=pubmed&cmd=Search&term="Carrizo+SJ"%5BAuthor%5D), [Vicente E](http://www.ncbi.nlm.nih.gov/entrez/query.fcgi?db=pubmed&cmd=Search&term="Vicente+E"%5BAuthor%5D), Agusti AG. Long-term cardiovascular outcomes in men with obstructive sleep apnoea-hypopnoea with or without treatment with continuous positive airway pressure: an observational study. *Lancet* 2005;**365**: 1046-1053.

[Gami AS](http://www.ncbi.nlm.nih.gov/entrez/query.fcgi?db=pubmed&cmd=Search&term="Gami+AS"%5BAuthor%5D), [Howard DE](http://www.ncbi.nlm.nih.gov/entrez/query.fcgi?db=pubmed&cmd=Search&term="Howard+DE"%5BAuthor%5D), [Olson EJ](http://www.ncbi.nlm.nih.gov/entrez/query.fcgi?db=pubmed&cmd=Search&term="Olson+EJ"%5BAuthor%5D), Somers AK. Day-night pattern of sudden death in obstructive sleep apnea. *N Engl J Med* 2005;**352**:1206-1214.

Masa JF, Rubio M, Findley LJ. Habitually sleepy drivers have a high frequency of automobile crashes associated with respiratory disorders during sleep. *Am. J. Respir. Crit. Care Med.* 2000; **162**: 1407-1412.

Young T, Palta M, Dempsey J *et al*. The occurrence of sleep-disordered breathing among middle-aged adults. *N. Engl. J. Med.* 1993; **328**: 1230-5.

Bearpark H, elliot L, Grunstein R. *et al.* Snoring and sleep apnea: a population study in Australian men. *Am. J. Respir. Crit. Care Med.* 1995; **151**: 1459-65.

Ip MS, Lam B, Lauder I *et al*. A community study of sleep-disordered breathing in middle-aged Chinese men in Hong Kong. *Chest* 2001; **119**: 62-9.

Bixler EO, Vgontzas AN, Ten Have T, *et al*. Effects of age on sleep apnea in men. *Am. J. Respir. Crit. Care Med.* 1998; **157**: 144-8.

Young T, Shahar E, Nieto JF, *et al*. Predictors of sleep-disordered breathing in community-dwelling adults: the Sleep Heart Health Study. *Arch. Intern. Med.* 2002; **162**: 893-900.

Ancoli-Isreal S, Kripke DF, Klauber MR, *et al.* Sleep-disordered breathing in community-dwelling elderly. Sleep 1991; **14(6)**: 486-95.

Young T, Palta M, Dempsey J, *et al.* The occurrence of sleep-disordered breathing among middle-aged adults. *N. Engl. J. Med.* 1993;**328**: 1230-5.

Feinsilver SH, Hertz G.. Sleep in the elderly patient. *Clin. Chest Med.* 1993;**14**:405-11.

Hume KI, Van F, Watson A. A field study of age and gender differences in habitual adult sleep. *J. Sleep Res.* 1998; **7**:85-94.

Ancoli-Isreal S, Klauber MR, Kripke DF, et al. Sleep apnoea in female patients in a nursing home. *Chest* 1989; **96**: 1054-8.

Ancoli-Isreal S, Kripke DF, Klauber MR, et al. Morbidity, mortality and sleep-disordered breathing in community dwelling elderly. *Sleep* 1996; **19**: 275-6.

Tabloski PA. Global aging: implications for women and women’s health. *J. Obstet. Gynecol. Neonatal Nurs.* 2004; **33**: 627-638.

Kump K, Whalen C, Tishler P *et al*. Assessment of the validity and utility of a sleep-symptom questionnaire. *Am. J. Respir. Crit. Care Med.* 1994; **150**: 735-41.

Johns MW. A new method for measuring daytime sleepiness: the Epworth sleepiness scale. *Sleep* 1991; **14**: 540-5

Walters AS: Toward a better definition of the restless legs syndrome. The International Restless Legs Syndrome Study Group. *Mov. Disord.* 1995;10:634-642.

Crocker BD, Olson LG, Saunders NA, Hensley MJ, Mckeon JL, Allen KM, Gyulay SG: Estimation of the probability of disturbed breathing during sleep before a sleep study. *Am. Rev. Respir. Dis.* 1990; **142**: 14-18.

Kapuniai L, Andrew D, Crowell D, Pearce J: Identifying sleep apnea from self-reports. *Sleep* 1998; **11**: 430-436.

Olson LG, King MT, Hensley MJ, Saunders NA: A community study of snoring and sleep disordered breathing: Symptoms. *Am. J. Respir. Crit. Care Med.* 1995; **152**: 707-710.

Hui DS, Chan J, Choy D *et al.* Effects of augmented CPAP education and support on compliance and outcome in a Chinese population. *Chest* 2000; **117**: 1410-6.

Flemnons W, Reimer MA. Development of a disease specific health-related quality of life questionnaire for sleep apnea. *Am. J. Respir. Crit. Care Med.* 1998; **158**: 494-503.
